# Advantages of meta-total RNA sequencing (MeTRS) over shotgun metagenomics and amplicon-based sequencing in the profiling of complex microbial communities

**DOI:** 10.1038/s41522-017-0046-x

**Published:** 2018-01-18

**Authors:** Fabien Cottier, Kandhadayar Gopalan Srinivasan, Marina Yurieva, Webber Liao, Michael Poidinger, Francesca Zolezzi, Norman Pavelka

**Affiliations:** 1 Singapore Immunology Network (SIgN), Agency for Science, Technology and Research (A*STAR), 8A Biomedical Grove, Immunos #04, Singapore, 138648 Singapore; 2 Present Address: The Jackson Laboratory for Genomic Medicine, Farmington, CT 06032 USA; 3 Present Address: GALDERMA R&D, Sophia Antipolis, Cedex 06902 France

## Abstract

Sequencing-based microbiome profiling aims at detecting and quantifying individual members of a microbial community in a culture-independent manner. While amplicon-based sequencing (ABS) of bacterial or fungal ribosomal DNA is the most widely used technology due to its low cost, it suffers from PCR amplification biases that hinder accurate representation of microbial population structures. Shotgun metagenomics (SMG) conversely allows unbiased microbiome profiling but requires high sequencing depth. Here we report the development of a meta-total RNA sequencing (MeTRS) method based on shotgun sequencing of total RNA and benchmark it on a human stool sample spiked in with known abundances of bacterial and fungal cells. MeTRS displayed the highest overall sensitivity and linearity for both bacteria and fungi, the greatest reproducibility compared to SMG and ABS, while requiring a ~20-fold lower sequencing depth than SMG. We therefore present MeTRS as a valuable alternative to existing technologies for large-scale profiling of complex microbiomes.

## Introduction

Conceptually, meta-total RNA sequencing (MeTRS) consists of three critical steps: (i) a protocol to extract total RNA with equal efficiency from both fungal and bacterial cells, (ii) a protocol to prepare RNA-sequencing libraries compatible with long paired-end Illumina reads and (iii) a bioinformatic pipeline to assign sequences at different taxonomic levels depending on their specificity. For RNA extraction, we tested several published protocols and commercial kits, and concluded that the classical hot-phenol extraction method provided the highest RNA yield from stool samples (Table S1), with little to no bias against bacteria or fungi when starting from artificial microbial communities (Table S2). We noticed that gut microbiome samples contain an unknown inhibitor that hindered subsequent library preparation steps (Table S1), but we solved this issue by treating the extracted RNA with a proprietary buffer from a commercial kit (Power Microbiome kit, MoBio, buffer PM1 and PM2). For sequencing library preparation, we found commercially available RNA-sequencing kits, which are optimized for generating relatively short fragment libraries and thus short sequencing reads, to yield sequences that lack sufficient uniqueness for unambiguous taxonomic assignment of most reads at the genus or species level. We performed in silico simulations and found that sequences should be at least 300 bp in length for accurate taxonomic assignment (Fig. S1), which could be accommodated by the setup of a customized RNA-sequencing library preparation protocol and a partially overlapping 2 × 250 bp paired-end sequencing run (see Methods for details). Finally, for the bioinformatics pipeline, we realized that existing software for shotgun metagenomics (SMG) or amplicon-based sequencing (ABS) data analysis, such as MetaPhlAn^1^ or QIIME,^2^ was not suitable for MeTRS. Specifically, while metagenomics analysis tools such as MetaPhlAn intentionally ignore ribosomal RNA (rRNA) sequences that represent the majority of MeTRS reads (Fig. S2), ABS pipelines such as QIIME implicitly assume that the sequences are derived from hypervariable rRNA regions and perform a pseudorandom taxonomic assignment when reads are rather derived from more conserved rRNA regions (Fig. S3).

To specifically handle the unique aspects of MeTRS data, we therefore developed a customized analysis pipeline (Fig. S2a) that first joins read pairs into longer pseudoreads and then maps each quality-filtered pseudoread based on stringent sequence similarity thresholds against a full-length, curated rDNA sequence database, such as SILVA,^3^ which contains sequences from all domains of life. Pseudoreads are then assigned to a taxonomy by a Consensus Taxonomy Tool (ConTxT): reads mapping to a single SILVA entry are directly assigned to the taxonomy of the corresponding entry; sequences matching more than one entry are subjected to an iterative algorithm, in which taxonomies associated to all matching entries are first compared at the lowest possible taxonomic level (e.g., species) and the taxonomic term found above a user-defined frequency threshold (currently defaulting to 60%) is assigned to the read; if no taxonomic term passes this threshold, the analysis is repeated at the next-highest taxonomic level (e.g., genus) and so on, until a level is found where a taxonomic term passes the threshold. Using this algorithm, we found, as anticipated, that 100% of the pseudoreads that successfully map to SILVA could be assigned at least at the domain level (i.e., one of bacteria, archaea or eukarya). Similarly to 16S sequencing, >80% of these reads could be assigned at the genus level (Fig. S1b).

Since one of MeTRS’ primary aims was to accurately report fungal in addition to bacterial composition in complex microbiome samples, we benchmarked the MeTRS method against the current standard for sequencing-based mycobiome profiling. While several different internal transcribed spacer (ITS) primers have been reported for this purpose, we used improved sequences of published ITS primers,^4^ which displayed a higher sensitivity of detection to a wide variety of fungi (Table S3 and Fig. S4).

## Results

To compare the different methods, we prepared a benchmarking sample set consisting of a healthy human donor stool homogenate, which was spiked in with six microbial species (three bacteria and three fungi) at six different concentrations (ranging from 10^4^ to 10^9^ cells per gram of stool) according to a Latin square design (Fig. S5). Total genomic DNA (gDNA) and RNA were then extracted in parallel from each sample, and while gDNA was analyzed by SMG, 16S or ITS ABS, total RNA was analyzed by MeTRS. This allowed us to rigorously assess the accuracy, sensitivity, linearity and reproducibility of each method with respect to profiling the microbiome composition of a well-defined complex microbial community.

**Fig. 1:**
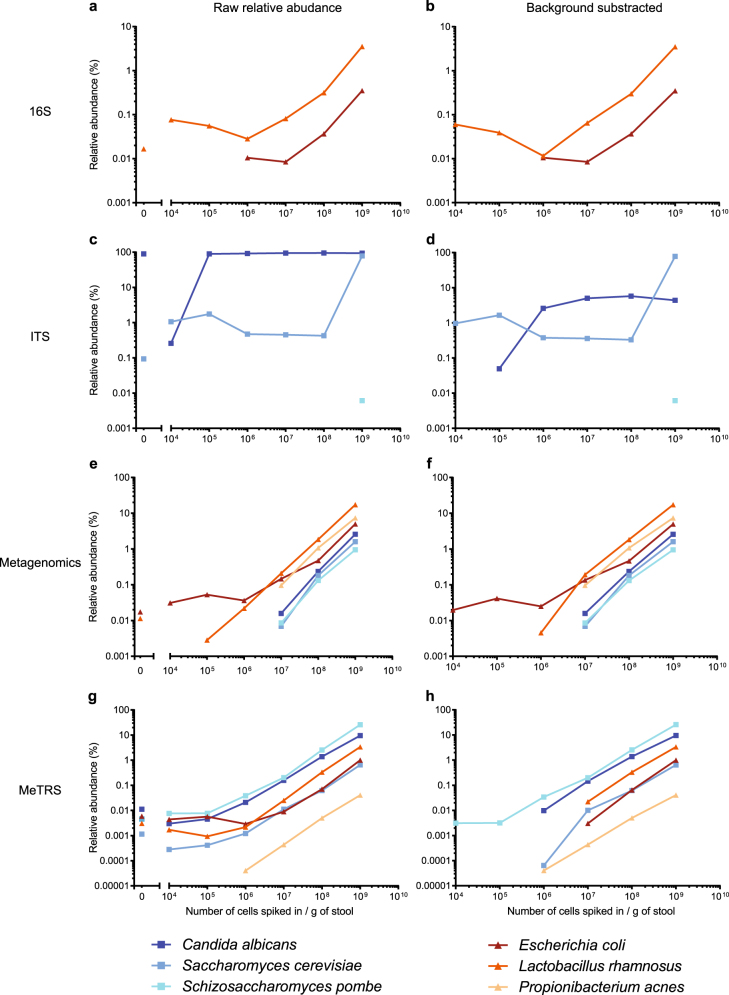
Performance comparison of microbiome profiling methods on Latin square spike-in data set. Relative abundances of yeast (*C. albicans*, *S. cerevisiae*, *S. pombe*) and bacteria (*E. coli*, *L. rhamnosus*, *P. acnes*) species are plotted as a function of the number of cells that were spiked into the background stool homogenate prior to DNA or RNA extraction (**a**, **c**, **e** and **g**). Relative abundances in the background sample were then subtracted from all other samples (**b**, **d**, **f** and **h**)

As expected, 16S and ITS ABS displayed high sensitivity towards four of the six species even at the lowest spiked-in concentrations of 10^4^ cells per gram of stool (Fig. 1a–d). *Propionibacterium acnes* was not detected in any 16S sample, and *S. pombe* was only detected in the ITS sample with the highest spiked-in concentration of 10^9^ cells per gram of stool, demonstrating clear polymerase chain reaction (PCR) biases that are attributable to primer sequence specificities. Moreover, ABS did not return a linear relationship between the spiked-in concentrations and the recovered relative abundances. This was especially the case during ITS sequencing, where relative abundances of *Candida albicans* and *Saccharomyces cerevisiae* displayed an all-or-nothing response, depending on which of the two species was spiked in at the higher concentration in the sample (Fig. 1c–d). As expected, SMG provided a more linear response in comparison to ABS, but suffered from a lack of sensitivity. With the exception of *E. coli* and *Lactobacillus rhamnosus* (which were already detected in the background sample), bacteria and fungi could only be detected when spiked-in at ≥10^7^ cells per gram of stool (Fig. 1e–f). MeTRS, on the other hand, was the only technology that detected in all samples all five species that are expected to be present in human stool (Fig. 1g–h). *P. acnes*, which is a skin and not a gut commensal, could be detected at spiked-in concentrations as low as 10^6^ cells per gram of stool, i.e., an order of magnitude lower than the detection limit of most species in SMG samples. This suggests that MeTRS exhibits a linear response over a wider dynamic range in comparison to the other methods.

Interestingly, we noticed MeTRS is superior to SMG with regards to detection of fungi. While SMG was unable to report any fungi in the background sample, MeTRS reported relative abundances of fungal species summing up to ~0.2% (Fig. 1e, g). Even at the highest spike-in concentrations, SMG consistently underrepresented fungal relative abundances (Fig. 1e–f), while in the case of MeTRS fungal relative abundances were at least as high as those of bacterial species (Fig. 1g–h). We confirmed by qRT-PCR that, in comparison to ribosomal DNA, fungal rRNA is relatively more abundant than bacterial rRNA on a per cell basis (Fig. S6), which is consistent with a larger cell size and higher ribosome content of fungi over bacteria.^5,6^

**Fig. 2:**
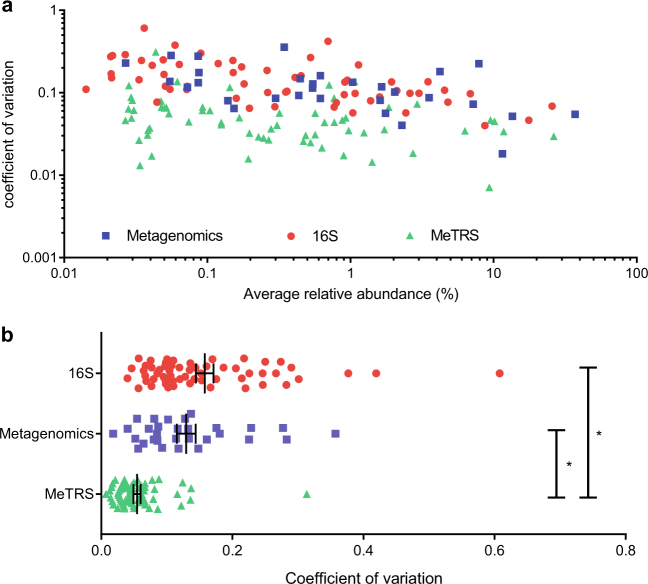
Comparison of the reproducibility of 16S sequencing, SMG and MeTRS. **a** For each genus that was detected in all seven samples (30 for SMG, 58 for 16S and 64 for MeTRS), a coefficient of variation (CV) was calculated for each sequencing method. CVs are plotted as a function of the average relative abundance of the corresponding genus across the seven samples. **b** Unpaired t-test with Welch’s correction was performed on these CVs. Error bars represent standard errors of the mean. *p < 0.0001

Assessing the 39 genera commonly detected in the un-spiked (background) stool sample by SMG, 16S and MeTRS, we noted that the MeTRS microbiome profile displayed significant similarity with both 16S and SMG approaches (Fig. S7). This confirms MeTRS as an appropriate method to profile microbiome communities. Focusing on genera detected at a relative abundance of ≥0.01%, we next analyzed the reproducibility of the relative abundances obtained for the non-spiked-in genera that were consistently detected in all seven samples by each method (30 for SMG, 58 for 16S sequencing and 64 for MeTRS). Surprisingly, relative abundances returned by MeTRS were significantly more reproducible than either SMG or 16S sequencing across the entire dynamic range of abundances (Fig. 2).

**Fig. 3:**
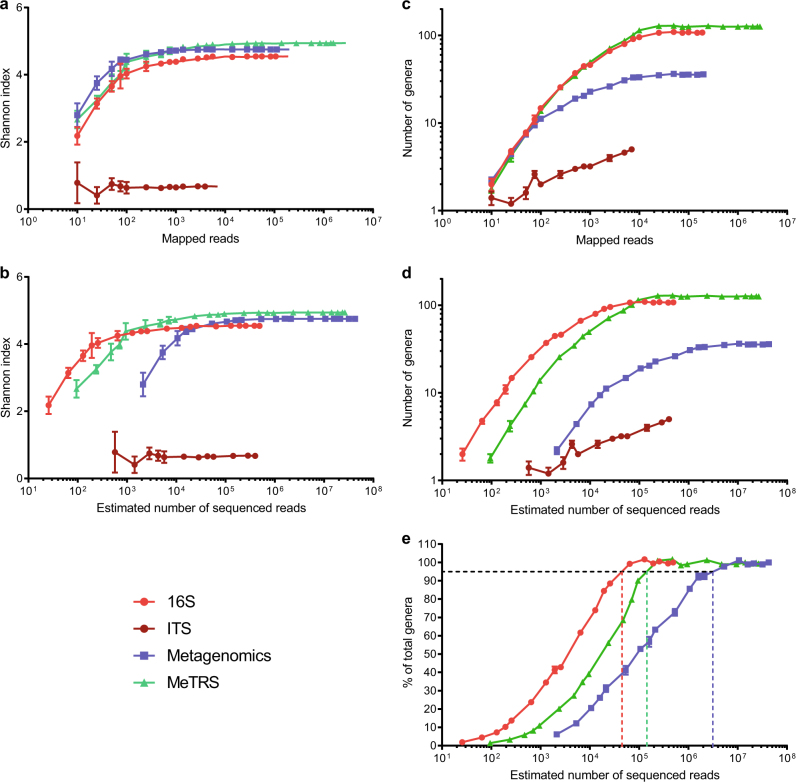
Sequencing cost comparison of microbiome profiling methods. Mapped reads from the background sample of the Latin Square spike-in data set were randomly subsampled at progressively larger fractions of the original data. Shannon indices (**a**, **b**) or number of unique genera (**c**, **d**) were calculated as an average of five independent random samples. Error bars represent standard deviations. Data are plotted as a function of either the number of mapped reads (**a**, **c**) or the estimated number of sequenced reads required to obtain that number of mapped reads (**b**, **d**). **e** Rarefaction data for 16S sequencing, MeTRS and metagenomics from panel **d** were re-plotted as a percentage of the maximum number of distinct genera identified at the highest sequencing depth. Dashed lines represent estimated sequencing depth required to detect 95% of those total genera

Finally, we evaluated the cost of each method in terms of depth of sequencing required to achieve close-to-saturation diversity and richness, by performing a rarefaction analysis of the non-spiked-in background sample. For this analysis, we focused on genera detected by ≥2 reads and associated with a relative abundance of ≥0.01%. With ~10^4^ mapped reads, MeTRS achieved a similar or higher α-diversity compared with SMG, and with ~2 × 10^4^ mapped reads it detected a similar or higher number of genera as 16S sequencing (Fig. 3a, b). Factoring in the vastly different mapping rates associated with each method (0.5% for SMG, 38.8% for 16S, 1.8% for ITS and 10.6% for MeTRS) (Table S4), MeTRS consistently outperformed SMG in terms of the estimated number of raw sequenced reads required to obtain the above-mentioned results (Fig. 3c, d). With the exception of ITS sequencing, all methods reached saturation in both diversity and richness, confirming that samples were indeed sequenced at a higher-than-required depth. Based on rarefaction analysis, we estimate that, in order to reach 95% saturation in genera richness (normalized to each method’s own estimated saturation level), at least ~3 × 10^6^ reads would be required for SMG, whereas ~50,000 would be sufficient for 16S sequencing. In comparison, ~150,000 reads would be needed for MeTRS to achieve the same endpoint, which is only ~3-fold higher than 16S sequencing but ~20 times lower than SMG. This could be explained by the fact that, in spite of the absence of any enrichment, depletion or PCR amplification step, MeTRS reads preferentially map to ribosomal small subunit (SSU, i.e., 16S or 18S) and large subunit (i.e., 23S or 25–28S) regions (Fig. S3), which are well known for their high taxonomic value. Bioinformatic tools have been developed to extract SSU-rRNA reads from SMG data,^7,8^ but these represent only a small fraction of a typical SMG sequencing run and are usually ignored by other software (e.g., MetaPhlAn).

## Discussion

All microbiome sequencing methods present advantages and downfalls.^9–14^ Metagenomics provides high accuracy but at a high cost and low sensitivity. Oppositely, amplicon sequencing does not allow comparison of different kingdoms simultaneously but is cost-effective and requires limited amount of starting material. Unfortunately, this method is also very sensitive to PCR biases and rRNA copy numbers. MeTRS has general limitations associated with working with RNA (RNA instability; cost and complexity of reverse transcription reaction; etc.), but these are not unlike standard RNA-sequencing for transcriptomics. The appropriateness of using RNA for the characterization of microbial communities has been debated as it is assumed that RNA content is linked more to cell physiology than to cell number, but no consensus on this question has been reached so far.^15^ Moreover, while MeTRS could potentially face problems in determining the exact relative abundance of species in a complex sample, it could be useful for the differentiation between live and dead cells or between metabolically more active and less active cells. Preliminary experiments seem to support this hypothesis (Table S2 and unpublished observation). Finally, MeTRS allows direct comparison of organisms from all kingdoms of life, albeit at a greater sensitivity and a lower cost than SMG.

In conclusion, we demonstrate that MeTRS (i) simultaneously detects both bacteria and fungi (ii) is overall more sensitive than SMG with a particular advantage in terms of fungal detection, (iii) achieves higher reproducibility than SMG or ABS, and (iv) requires significantly lower sequencing depth than SMG. For these reasons, we recommend MeTRS for profiling complex communities that consists of bacteria, fungi and possibly other microbes. We envisage that MeTRS will be a valuable tool for population-wide association studies in humans and possibly other large-scale environmental microbiome profiling studies. Moreover, the data sets generated in this study, namely, the Latin square stool spike-in and the 16-fungal-species mock community, will enable the development and benchmarking of bioinformatic pipelines for a variety of microbiome analysis applications.

## Methods

### Preparation of artificial fungal community

Sixteen different fungal species were grown in media specified in Table S3 until stationary phase was reached. Cells were collected and gDNA extracted as described below. Individual DNA concentrations were measured by Quant-iT PicoGreen dsDNA Assay Kit (ThermoFisher). An equimolar mixture of gDNA molecules from each species was prepared taking into account the respective genome sizes.

### Latin square stool spike-in sample preparation

Human stool sample was obtained with informed consent according to protocols approved by the National University of Singapore (NUS) Institutional Review Board (IRB) filed under NUS-IRB Reference Code 12-208 (Approval Number: NUS 1615). Thirteen grams of feces from a single donor was homogenized in cold PBS and filtered through a 70 μm filter. Aliquots equivalent to 0.5 g of feces per tube were prepared and stored at −80 °C.

*Candida albicans* (SC5314) was grown in yeast extract peptone dextrose (YPD) medium (1% w/v yeast extract, 2% w/v peptone and 2% w/v d-glucose, supplemented with 1.5% w/v agar for solid media only) at 37 °C, *Saccharomyces cerevisiae* (BY4741) in YPD medium at 30 °C and *Schizosaccharomyces pombe* (972 h) in Yeast extract-Malt extract medium (0.3% yeast extract, 0.3% malt extract, 1% dextrose, 0.5% peptone) at 30 °C. *Escherishia coli* (MG1655) was grown in Lysogeny Broth (1% tryptone, 0.5% yeast extract, 1% NaCl) at 37 °C, *Lactobacilus rhamnosus* GG in De Man, Rogosa and Sharpe medium^16^ (Sigma) at 37 °C, and *Propionibacterium acnes* was grown in BBL Schaedler Broth (BD) in a fermentor (New Brunswick) under anaerobic conditions (10% CO_2_, 90% N_2_) at 37 °C agitated at 50 rpm. Otherwise specified cells were cultured in a shaking incubator at 150 rpm. Once logarithmic growth phase was reached, cells were centrifuged at 3,500 rpm for 5 min and re-suspended in PBS. Cell concentrations were determined with a hemocytometer, adjusted to 1 × 10^10^ cells/ml and kept at −80 °C. Spike-ins were performed according to Fig. S3. Stool homogenate aliquots were spiked in with the appropriate number of cells, then half of the solution was used for DNA extraction, the other half for RNA extraction.

### DNA and RNA extraction

DNA was extracted according to the protocol described by Rancati et al.^17^ RNA extraction was performed according to manufacturer’s protocol (RNeasy and RNeasy PowerSoil from Qiagen; and Soil/Fecal RNA Kit from Zymoresearch; Power Microbiome Kit from MoBio) and as described by Pavelka et al.,^18^ with the following modifications. From the final 500 μl of total RNA suspension, 50 μl were treated with DNase (New England Biolabs) for 30 min at 37 °C. This was followed by treatment with buffer PM1 and PM2 from the Power Microbiome kit (MoBio) according to the manufacturer’s protocol. After this step of RNA cleaning, RNA precipitation was performed in a similar manner as the protocol from Pavelka et al.^18^

### 16S and ITS ABS

For amplification of the 16S variable regions (V4-V5), PCR was performed using 10 ng of gDNA with LongAmp Taq DNA polymerase (New England Biolabs) according to manufacturer’s instructions. Identification of fungal populations was carried out by amplifying the ITS2 region from 10 ng of gDNA template using Phusion High-Fidelity DNA Polymerase (ThermoFisher Scientific) as recommended by manufacturers. Primer sequences and other details can be found in Table S3.

The library preparation steps for both 16S and ITS2 regions were as follows. The reaction mix for 16S primary PCR contains a specific forward primer (V4 F) and reverse primer (V5 R) binding to V4–V5 regions. Similarly, another set of primers binding to the 5.8S (fITS7 or ITSf) and 25-28S rDNA regions (ITS4) was used for amplifying the ITS2 fragment. Primary PCR cycling parameters for amplifying 16S consisted of initial denaturation step for 30 s at 94 °C, followed by 15 cycles of 15 s at 94 °C, 30 s at 45 °C, and 30 s at 65 °C with a final extension for 10 min at 65 °C for the primary PCR reaction. ITS2 sequences were amplified by initial denaturation for 30 s at 98 °C, followed by 15 cycles of 15 s at 98 °C, 30 s at 55 °C, and 30 s at 72 °C with a final extension for 10 min at 72 °C. For secondary PCR, the cycling parameters were the same as described above for 16S and ITS primary PCR, respectively, except that amplification was carried out for 25 cycles for 16S and 30 cycles for ITS2 regions, respectively. During the secondary PCR reaction, barcodes for identifying and de-multiplexing individual samples, and Illumina adapter sequences, were added to the template using communal primers (Table S5).

Equimolar concentrations of secondary PCR products were pooled and electrophoresed using 1% agarose gel. Pooled amplicon libraries were gel-purified using the Qiaquick Gel Extraction Kit (Qiagen). Concentrations of gel-purified libraries were estimated using the DNA 1000 kit (Agilent Technologies).

### SMG sequencing

Genomic DNA (1 µg) was sheared using Covaris S2 sonicator in 52.5 μl volume using the following parameters: 10% duty cycle, intensity 4 and 200 cycles per burst for 120 s. Sequencing libraries were prepared according to a modified version of a previously published protocol.^19^ Briefly, instead of performing a clean-up step after enzymatic treatment of the DNA sample, we performed heat inactivation after end-repair, dA-tailing, and ligation. For processing, 350 ng of fragmented gDNA in a volume of 17 µl were used for the library preparation. NEXTflex DNA barcodes (Bioo Scientific) were added for multiplexing and sequencing the libraries.

Adapter-ligated DNA was purified and size-selected using Agencourt AMPure XP beads (Beckman Coulter). Clean-up was performed after adjusting the volume of reaction mix after adapter ligation to 55 µl. AMPure beads were added at 1:1 ratio and the cleaned-up DNA was eluted in 50 µl of re-suspension buffer. Eluted DNA was repurified using AMPure beads added at 1:1 ratio. Finally the double cleaned-up, adapter-ligated DNA was eluted in 20 µl of re-suspension buffer.

Using 20 µl of eluted library, PCR was performed in a 50 µl volume containing 1× Phusion Master Mix with HF Buffer (ThermoFisher Scientific) and Illumina PE 1.0 and 2.0 primers (Bioo Scientific). PCR conditions consisted of initial denaturation of 1 min at 98 °C followed by ten cycles of 30 s at 98 °C, 30 s at 65 °C, 30 s at 72 °C, followed by an extension of 10 min at 72 °C. Post-PCR clean-up of library was performed using 1:1 ratio of AMPure XP beads (Beckman Coulter). The libraries were re-suspended in 30 µl of re-suspension buffer. Concentrations of the purified libraries were estimated using the DNA 1000 kit (Agilent Technologies).

### Total RNA sequencing

Double-stranded cDNA was prepared from 2 µg of total RNA extracted from stool samples using Superscript^®^ double stranded cDNA synthesis kit (ThermoFisher Scientific) according to manufacturer’s instructions, except that the first strand cDNA synthesis was primed using random hexamers (Promega). Double stranded cDNA (200 ng) was sheared using Covaris S2 sonicator in 52.5 μl volume with the following parameters: 10% duty cycle, intensity 4 and 200 cycles per burst for 70 s. Subsequently, 17 µl of sheared cDNA (65 ng) was end-repaired, A-tailed, adapter-ligated, Ampure XP beads-purified and libraries were PCR-enriched as described above for metagenome library preparation. PCR-enriched libraries were cleaned up and size-selected to remove unused dNTPs, primers and short RNA fragments, using a 0.65 × ratio of AMPure XP beads (Beckman Coulter).

### High-throughput sequencing

All libraries were quantified using KAPA Library Quantification Kit (Kapa Biosystems) to ascertain the loading concentration and sequenced on a HiSeq 2500 System (Illumina) operated in Rapid Run Mode to generate 2 × 250 bp paired-end reads. Sequencing depths are listed in Table S2. All sequencing results have been deposited at NCBI Sequence Read Archive (SRA) under accession number SRP103706.

### Data analysis

The bioinformatics pipeline can be divided into two steps, consisting of the read pre-processing step and the OTU picking/taxonomy assignment step (Fig. S2a). During the read pre-processing step, sequencing adapters and PCR primers were first removed using the ILLUMINACLIP step in Trimmomatic^20^ (v0.35) run in paired-end mode. Paired-end reads were then joined using FLASh^21^ (v1.2.11) with a maximum overlap of 250 bp, before quality trimming (qtrim = rl; trimq = 30) using BBDuk of BBTools (v35.85) and finally filtered for at least 50 bp using the MINLEN step in Trimmomatic run in single-end mode. After preprocessing, 16S and ITS samples were mapped against the SILVA^3^ (v.123) and UNITE^22^ databases, respectively. For both pipelines, the pick_closed_reference_otus.py script in QIIME^2^ (v1.8.0), implementing the usearch^23^ (v7.0.1090) algorithm, was used for OTU picking, enabling reverse strand matching and requiring a minimum similarity of 99%. SMG samples were analyzed using MetaPhlAn2^24^ (v2.5.0) with default parameters. MeTRS samples were analyzed using the following in-house built pipeline.

The MeTRS pipeline is composed of two steps, the mapping step and the taxonomy assignment step. In the first step, the sample is mapped against the SILVA database (v.123) using Bowtie^25^ (v1.1.2) and the following parameters: -a -v1 --best --strata. Reads are then assigned to a consensus taxonomy of the mapped hits using an in-house built Python (v2.7) script, called Consensus Taxonomy Tool (ConTxT). Starting at the species level, if >60% of the mapped hits of a read agrees on the same taxonomic term, then that taxonomic term is assigned to the read. If no agreement is reached, information from the next highest taxonomic level is iteratively interrogated until a consensus is found. If no consensus is achieved even at the highest taxonomic level (i.e., domain), the read is assigned to the “unknown” domain. All relevant code is available at https://github.com/normanpavelka/MeTRS.

Within-sample microbial diversity (α-diversity) was estimated with the Shannon–Wiener diversity index^26^ as implemented in QIIME. To examine the effect of subsampling and to estimate the minimal required sequencing depth for each sequencing technology, rarefactions were performed on the mapped reads of all the background samples using QIIME with five iterations. The samples were rarefied at the same depths and, for the last rarefaction depth, all of the samples’ mapped reads rounding down to two significant digits (Table S6). A mean α-diversity index and an average number of OTUs across the five independent iterations were calculated for each sample at each depth. The estimated sequencing depth was calculated as a factor of the rarefaction depth and the ratio of the number of mapped reads to the number of sequenced reads.

### Quantitative RT-PCR

Double-stranded cDNA was synthesized starting from ~100 ng of total RNA using the Superscript III kit (Invitrogen) in 20 μl reaction volumes as per manufacturers’ protocol. Quantitative PCR reactions were then set up in 384-well plates and cycled using an ABI 7900HT (Applied Biosystems) in 10 μl reaction volumes using the following primers: FungiQuant-F and FungiQuant-R,^27^ or BactQuant-F and BactQuant-R.^28^ Fold changes were then computed according to the standard ΔCt method.^29^

### Data availability

All data that support the findings of this study are available from the corresponding author upon request. Raw sequencing data has been deposited at NCBI under SRA accession number SRP103706.

### Code availability

The code required for parsing the SILVA taxonomy and for running the Consensus Taxonomy Tool (ConTxT) can be found at https://github.com/normanpavelka/MeTRS

## Electronic supplementary material

Supplementary Information
