# Supplementary material for: Advantages of meta-total RNA sequencing (MeTRS) over shotgun metagenomics and amplicon-based sequencing in the profiling of complex microbial communities
Source: NPJ Biofilms Microbiomes. 2018 Jan 18;4:2. doi: 10.1038/s41522-017-0046-x (PMC5773663; doi:10.1038/s41522-017-0046-x)
Supplement: Supplementary file 1 — Supplementary Information [file 41522_2017_46_MOESM1_ESM.docx]

**Supplementary Table 1: Comparison of protocols used for RNA extraction from human stool.** We obtained sufficient amount of RNA with all methods, but extracts were not always suitable for MeTRS library construction.

|  | RNA extraction yield (μg RNA / g of stool) ^a^ | | |  |
| --- | --- | --- | --- | --- |
| **RNA extraction method** | **Donor 1** | **Donor 2** | **Donor 3** | **Library preparation** |
| Qiagen RNAeasy | 5 | 7 | n.t. | Unsuccessful |
| Qiagen RNeasy PowerSoil | n.t. | 10 | n.t. | Unsuccessful |
| Zymoresearch Soil/Fecal RNA Kit | n.t. | n.t. | 11 | Unsuccessful |
| MoBio Power Microbiome kit | 85 | 31 | 50 | Successful |
| Acid Phenol | 1935 | 1497 | 1632 | Inconsistent |
| Final MeTRS protocol | 559 | 370 | 453 | Successful |

Notes:
a) n.t. = not tested

**Supplementary Table 2: MoBio Power Microbiome kit displays a bias against fungi.** While the MoBio kit was efficient in extracting RNA with good quantity and quality, we observed that this method struggles to extract RNA from yeast. The Acid phenol protocol shows no such bias, but as demonstrated in Supplementary Table 1, the RNA does not always lead to successful library construction. Our final MeTRS protocol takes advantages of both methods.

|  | | | RNA extraction yield (μg RNA / million cells) | | |
| --- | --- | --- | --- | --- | --- |
| **Artificial mix ^a^** | | | **MoBio kit** | **Acid Phenol** | **Final protocol** |
|  |  |  |  |  |  |
| Mix 1 | 50% *C. albicans* log phase | 50% *L. rhamnosus* log phase | 6.815 | 56.175 | 8.8 |
| Mix 2 | 50% *C. albicans* log phase | 50% *L. rhamnosus* O/N culture | 0.44 | 26.55 | 2.075 |
| Mix 3 | 50% *C. albicans* O/N culture | 50% *L. rhamnosus* log phase | 5.51 | 34.675 | 2.325 |

Notes:
a) Total cell number in each mix: 2 × 10^6^ cells. Yeast or bacterial cells were either exponentially growing (log phase) or in stationary phase (O/N culture). Log-phase cells are expected to yield more total RNA, and more ribosomal RNA, per cell^1^.

**Supplementary Table 3. Strains used in this study**

| **Domain / Kingdom** | **Species name** | **Strain reference** | **Growth medium** |
| --- | --- | --- | --- |
| Fungi | *Candida albicans* | SC5314 | YPD |
| Fungi | *Candida parapsilosis* | GA1 | YPD |
| Fungi | *Candida glabrata* | ATCC 2001 | YPD |
| Fungi | *Candida tropicalis* | ATCC 13803 | YPD |
| Fungi | *Candida krusei* | ATCC 14243 | YPD |
| Fungi | *Candida dublinesis* | ATCC MYA-646 | YPD |
| Fungi | *Saccharomyces cerevisiae* | BY4741 | YPD |
| Fungi | *Schizosaccharomyces pombe* | 972h- | YM |
| Fungi | *Schizosaccharomyces japonicus* | ATCC10660 | YM |
| Fungi | *Aspergillus fumigatus* | ATCC MYA-4609 | YM |
| Fungi | *Pichia anomala* | ATCC 20029 | YM |
| Fungi | *Cryptococcus neoformans* | ATCC 66031 | YPD |
| Fungi | *Saccharomyces paradoxus* | ATCC 96966 | YPD |
| Fungi | *Trichoderma virens* | ATCC 52045 | YM |
| Fungi | *Phanerochaete chrysosporium* | ATCC 20696 | YM |
| Fungi | *Sclerotinia sclerotiorum* | ATCC 46762 | YM |
| Bacteria | *Escherichia coli* | MG1655 | LB |
| Bacteria | *Lactobacillus rhamnosus* GG | ATCC 53103 | MRS |
| Bacteria | *Propionibacterium acnes* | ATCC 6919 | BBL Schaedler |

**Supplementary Table 4. Sequencing data summary**

| **Tech ^a^** | **ID ^b^** | **Name ^c^** | **Library size ^d^** | **Sequenced reads (total)** | **Filtered reads (total)** | **Mapped reads (total)** | **Filtered reads (% of sequenced)** | **Mapped reads (% of sequenced)** |
| --- | --- | --- | --- | --- | --- | --- | --- | --- |
| MeTRS | RHH4367 | bg | 592 | 27,361,000 | 10,315,720 | 2,892,979 | 37.7% | 10.6% |
| MeTRS | RHH4368 | A | 569 | 47,835,005 | 15,873,888 | 6,418,465 | 33.2% | 13.4% |
| MeTRS | RHH4369 | B | 562 | 45,823,063 | 18,000,549 | 6,413,569 | 39.3% | 14.0% |
| MeTRS | RHH4370 | C | 567 | 40,637,232 | 15,341,080 | 4,992,660 | 37.8% | 12.3% |
| MeTRS | RHH4371 | D | 583 | 48,106,309 | 18,463,888 | 5,567,828 | 38.4% | 11.6% |
| MeTRS | RHH4372 | E | 581 | 47,246,680 | 17,484,398 | 5,145,088 | 37.0% | 10.9% |
| MeTRS | RHH4373 | F | 508 | 45,805,287 | 16,543,342 | 5,287,468 | 36.1% | 11.5% |
| SMG | DHH086 | bg | 474 | 43,316,068 | 9,602,459 | 202,145 | 22.2% | 0.5% |
| SMG | DHH087 | A | 390 | 42,597,813 | 8,793,296 | 176,768 | 20.6% | 0.4% |
| SMG | DHH088 | B | 459 | 51,935,766 | 14,188,723 | 288,728 | 27.3% | 0.6% |
| SMG | DHH089 | C | 473 | 61,333,686 | 20,004,363 | 412,835 | 32.6% | 0.7% |
| SMG | DHH090 | D | 434 | 42,603,595 | 13,578,132 | 283,863 | 31.9% | 0.7% |
| SMG | DHH091 | E | 457 | 43,317,038 | 15,021,088 | 329,930 | 34.7% | 0.8% |
| SMG | DHH092 | F | 493 | 46,714,062 | 15,786,657 | 349,712 | 33.8% | 0.7% |
| 16S | DHH100 | bg | 550  (pooled) | 496,760 | 372,581 | 192,806 | 75.0% | 38.8% |
| 16S | DHH101 | A |  | 382,300 | 299,923 | 162,992 | 78.5% | 42.6% |
| 16S | DHH102 | B |  | 425,477 | 334,078 | 178,132 | 78.5% | 41.9% |
| 16S | DHH103 | C |  | 445,507 | 292,169 | 145,348 | 65.6% | 32.6% |
| 16S | DHH104 | D |  | 276,638 | 182,642 | 94,687 | 66.0% | 34.2% |
| 16S | DHH105 | E |  | 235,507 | 129,054 | 63,432 | 54.8% | 26.9% |
| 16S | DHH106 | F |  | 256,460 | 88,980 | 35,321 | 34.7% | 13.8% |
| ITS | DHH114 | bg | 420  (pooled) | 426,888 | 15,426 | 7,481 | 3.6% | 1.8% |
| ITS | DHH115 | A |  | 743,542 | 17,537 | 15,342 | 2.4% | 2.0% |
| ITS | DHH116 | B |  | 450,483 | 16,604 | 6,207 | 3.7% | 1.4% |
| ITS | DHH117 | C |  | 276,251 | 9,145 | 3,772 | 3.3% | 1.4% |
| ITS | DHH118 | D |  | 1,109,986 | 26,289 | 21,753 | 2.4% | 2.0% |
| ITS | DHH119 | E |  | 488,585 | 15,038 | 11,496 | 3.1% | 2.4% |
| ITS | DHH120 | F |  | 627,968 | 36,918 | 16,348 | 5.9% | 2.6% |
| ITS | AMG099 | ICP |  | 435,740 | 351,527 | 283,250 | 80.7% | 65.0% |
| ITS | AMG100 | IPP |  | 463,838 | 373,171 | 276,042 | 80.5% | 59.5% |

Notes:
a) Technology: SMG = shotgun metagenomics
b) Sample ID as deposited in NCBI
c) Sample name: bg = Background; ICP = ITS Custom Primers; IPP = ITS Published Primers
d) Peak library size in bp as determined by Agilent Bioanalyzer. Includes 125 bp of adapters. All 16S samples were pooled prior to library size quantification; the same applies to ITS samples.

**Supplementary Table 5. Primers used in this study**

| **Name** | **Sequence ^a^** | **Reference** |
| --- | --- | --- |
|  |  |  |
| V4 F | 5’-ACACTCTTTCCCTACACGACGCTCTTCCGATCTCAGCMGCCGCGGTAAYWC-3’ | Modified from ^2^ |
| V5 R | 5’-GTGACTGGAGTTCAGACGTGTGCTCTTCCGATCTBSCCCGYCAATTYMTKTRAGT-3’ | Modified from ^3^ |
| fITS7 | 5’-ACACTCTTTCCCTACACGACGCTCTTCCGATCTGTGARTCATCGAATCTTTG-3’ | Adapted from ^4^ [Illumina adapters added] |
| ITSf | 5’-ACACTCTTTCCCTACACGACGCTCTTCCGATCTGTGARTCATCGARTYYTTG-3’ | This study [modified from fITS7] |
| ITS4 | 5’-GTGACTGGAGTTCAGACGTGTGCTCTTCCGATCTTCCTCCGCTTATTGATATGC-3’ | ^5^ |
| TruSeq Universal Adapter (forward) | 5’-AATGATACGGCGACCACCGAGATCTACACTCTTTCCCTACACGACGCTCTTCCGATCT-3’ | Illumina |
| TruSeq Indexed Adapter (reverse) | 5’-CAAGCAGAAGACGGCATACGAGATNNNNNNGTGACTGGAGTTC-3’ | Illumina |
| BactQuant-F | 5′-CCTACGGGDGGCWGCA-3′ | ^6^ |
| BactQuant-R | 5′-GGACTACHVGGGTMTCTAATC-3′ | ^6^ |
| FungiQuant-F | 5′-GGRAAACTCACCAGGTCCAG-3′ | ^6^ |
| FungiQuant-R | 5′-GSWCTATCCCCAKCACGA-3′ | ^6^ |

Notes:
a) NNNNNN represents the barcode sequence.

**Supplementary Table 6. Rarefaction depths**

| **Sequencing Technology** | **Sample** | **Number of sequenced reads** | **Number of mapped reads** | **Rarefaction Depths (number of sampled reads) ^a^** |
| --- | --- | --- | --- | --- |
| Metagenomics | DHH086 | 43,316,068 | 202,145 | 200k, 150k, 100k, 75k, 50k, 25k, 10k, 7.5k, 5k, 2.5k, 1k, 750, 500, 250, 100, 75, 50, 25, 10 |
| 16S | DHH100 | 496,760 | 192,806 | 190k, 150k, 100k, 75k, 50k, 25k, 10k, 7.5k, 5k, 2.5k, 1k, 750, 500, 250, 100, 75, 50, 25, 10 |
| ITS | DHH114 | 426,888 | 7,481 | 7k, 5k, 2.5k, 1k, 750, 500, 250, 100, 75, 50, 25, 10 |
| MeTRS | RHH4367 | 27,361,000 | 2,892,979 | 2.8m, 2.5m, 2m, 1.5m, 1m, 500k, 250k, 100k, 75k, 50k, 25k, 10k, 7.5k, 5k, 2.5k, 1k, 750, 500, 250, 100, 75, 50, 25, 10 |

Notes:
a) ‘m’ = ‘×10^6^’; ‘k’ = ‘×10^3^’


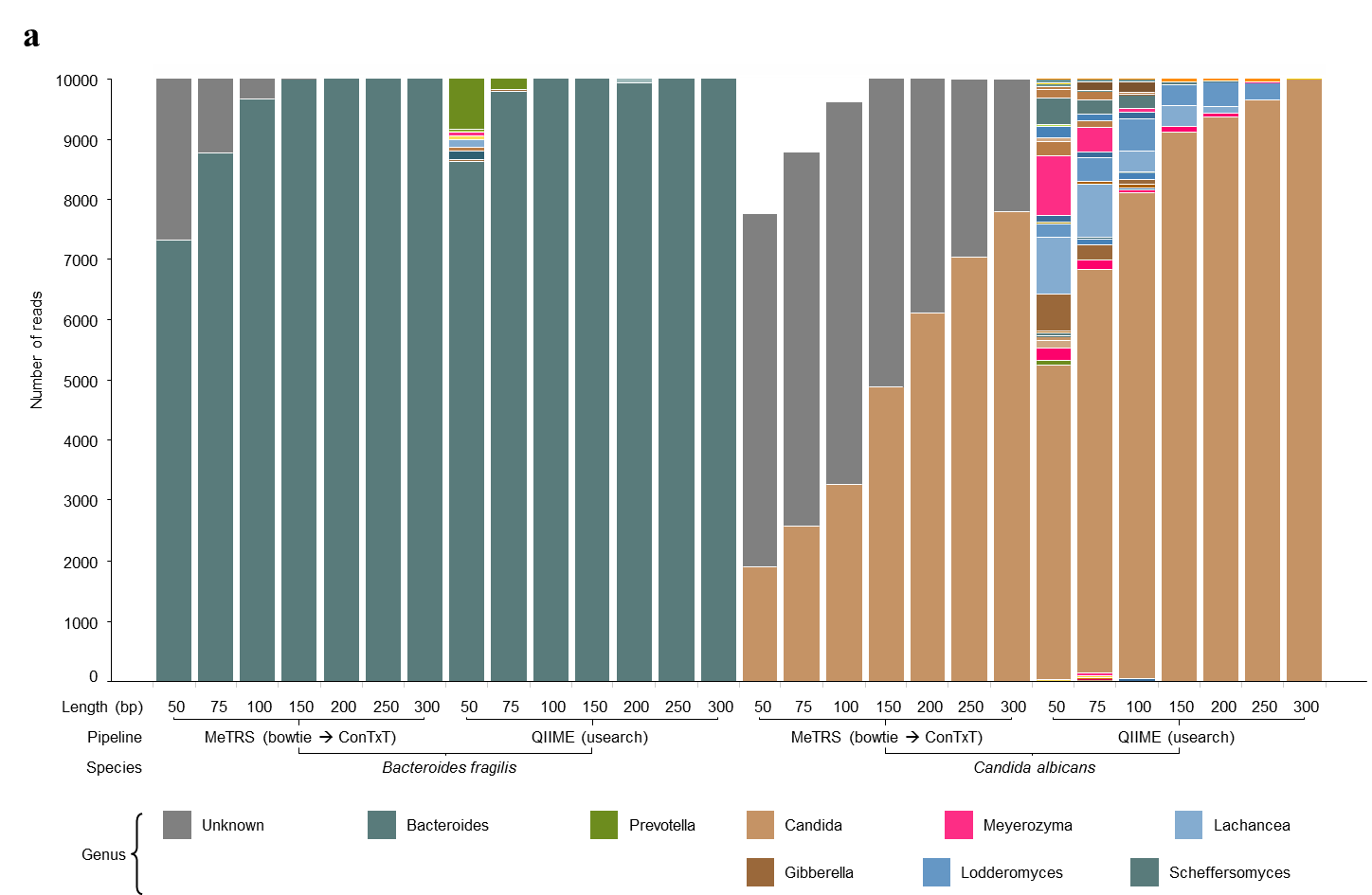


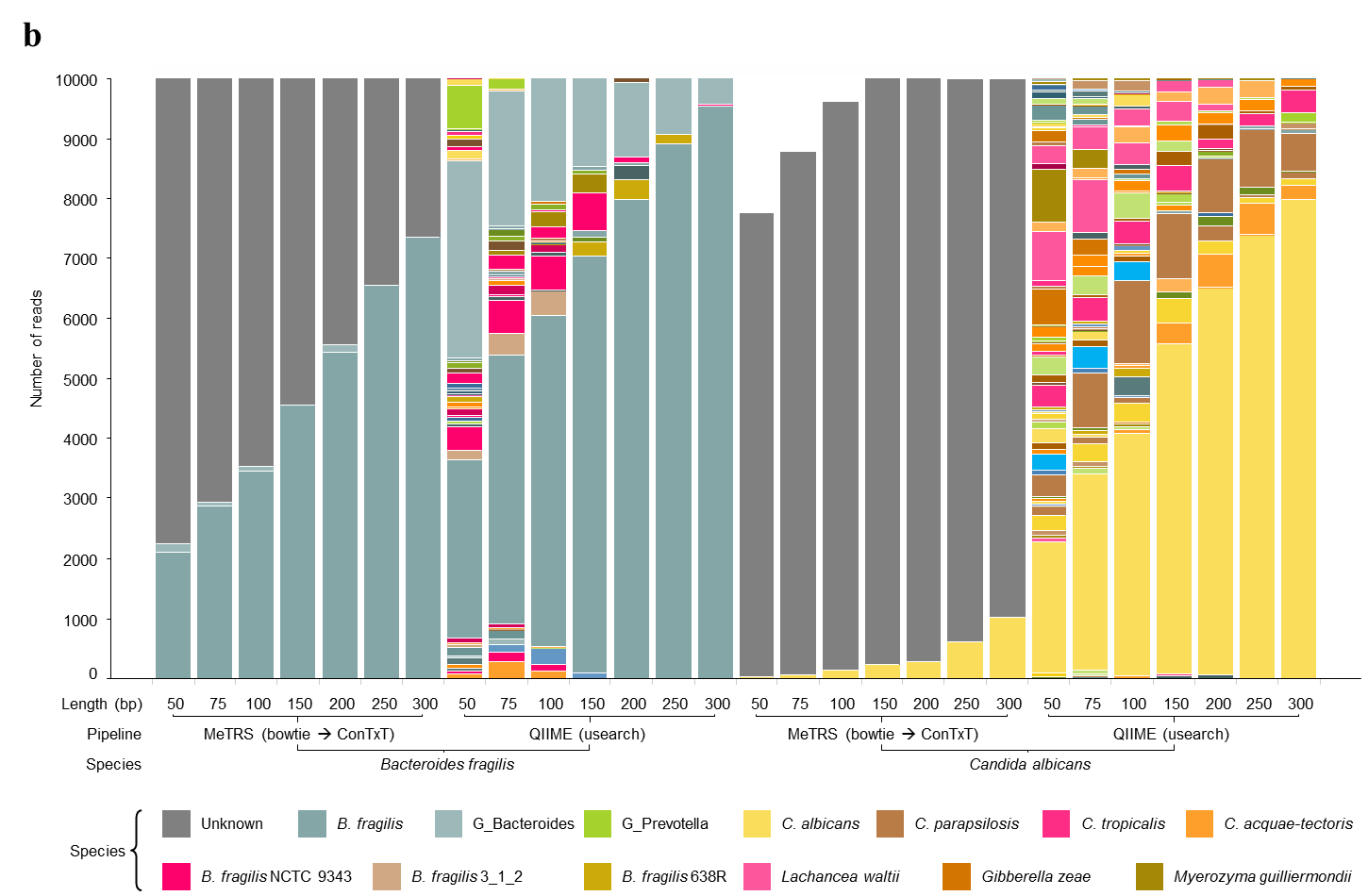


**Supplementary Figure 1. *In silico* simulation.**

Ten thousand random fragments of a particular length (ranging from 50 to 300 bp) were generated from full-length SSU rRNA sequences of the indicated species (either *B. fragilis* or *C. albicans*) and mapped against the SILVA SSU database using either the standard QIIME pipeline or the customized MeTRS pipeline. Plots show number of reads mapped at a particular genus (**a**) or species (**b**). G_xxx = undetermined species of genus xxx.


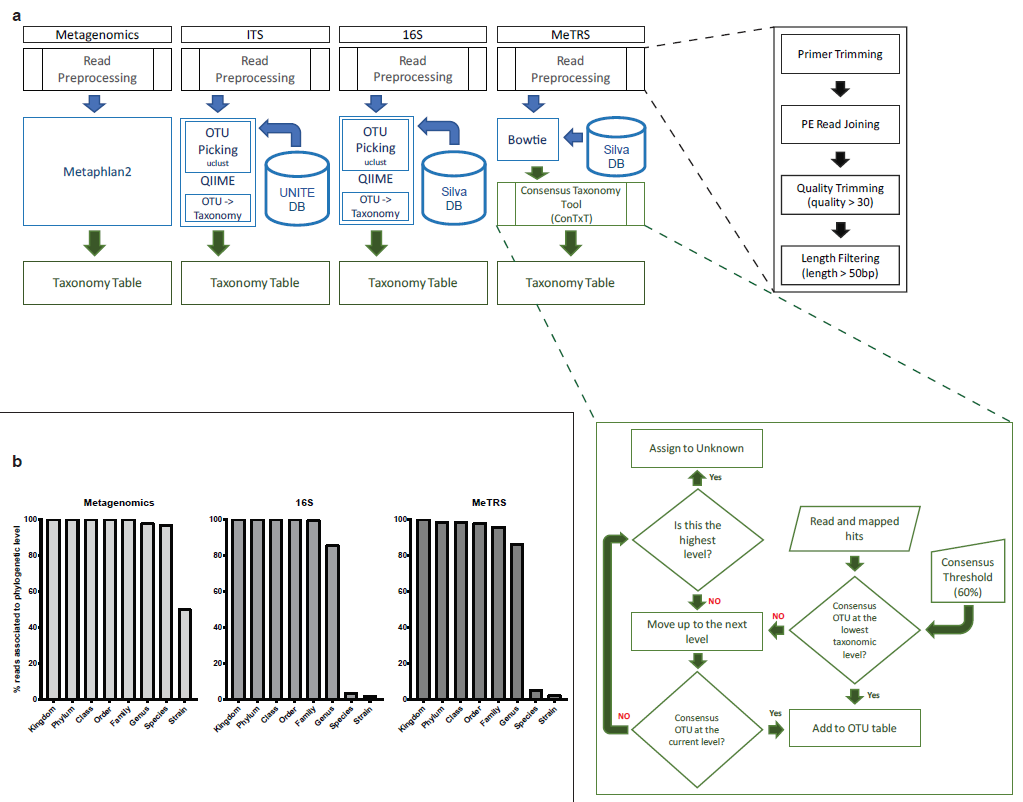


**Supplementary Figure 2. Data analysis pipeline.**

(**a**) Schematic representation of the analytical pipeline for metagenomics, ITS, 16S and MeTRS from reads processing to taxonomical identification table. (**b**) Percentage of reads directly assigned to each taxonomical level for metagenomics, 16S and MeTRS.

**
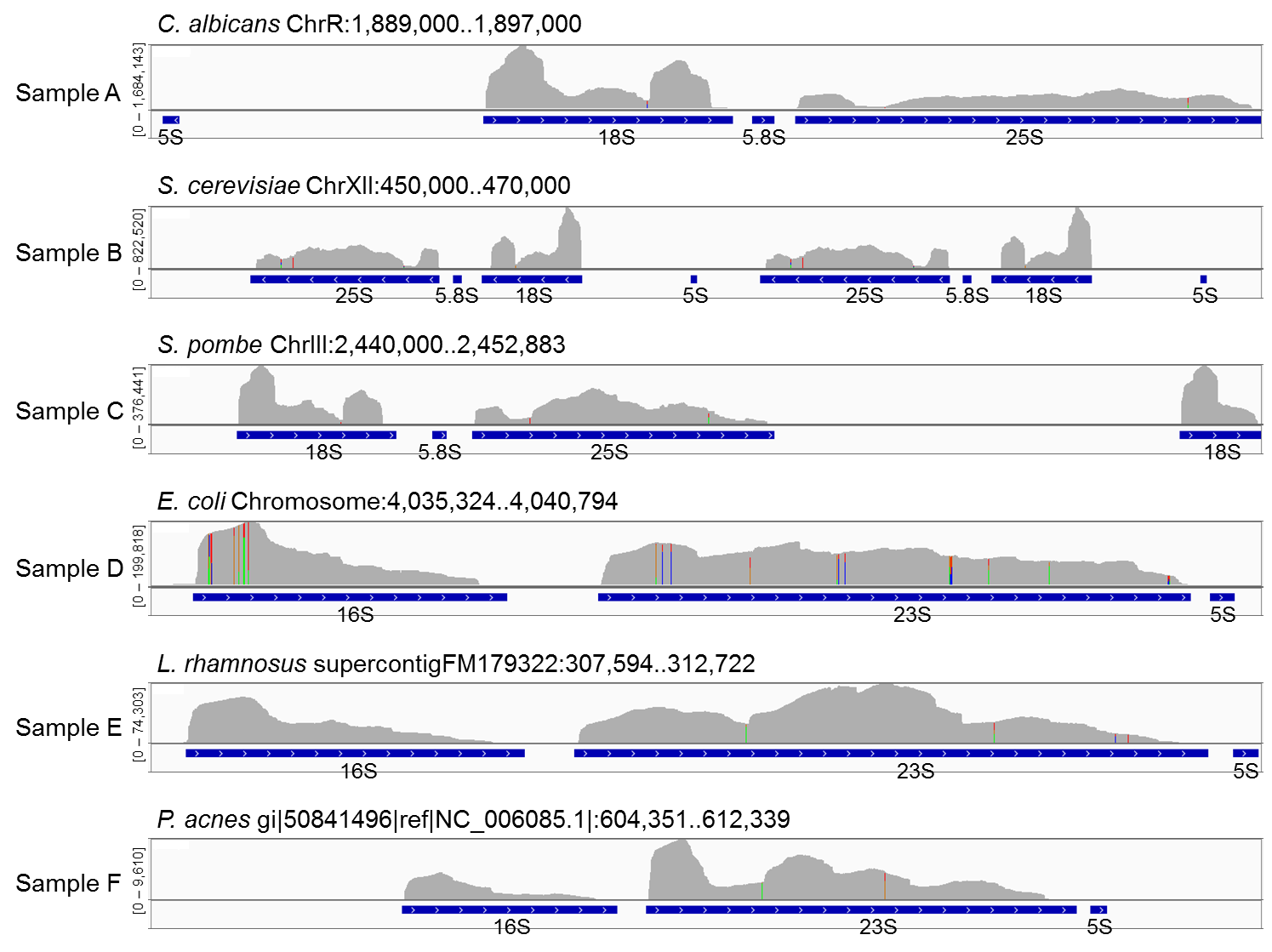
**

**Supplementary Figure 3. Mapping of MeTRS reads against the rRNA locus of spiked-in species.**

The six indicated MeTRS samples from the Latin Square stool spike-in dataset were mapped with bowtie against the indicated rRNA loci. Coverage plots were generated with Integrative Genomics Viewer v.2.4.1 and each graph was auto-scaled to its own data range, as indicated to the left of each plot. As expected, reads mapped almost exclusively to rRNA genes and not to intergenic regions. Moreover, reads were almost absent from 5S and 5.8S regions, as these are ~100 and ~150bp in length, respectively, while MeTRS libraries are typically >300 bp in size (Table S4) due to the size selection step.

**
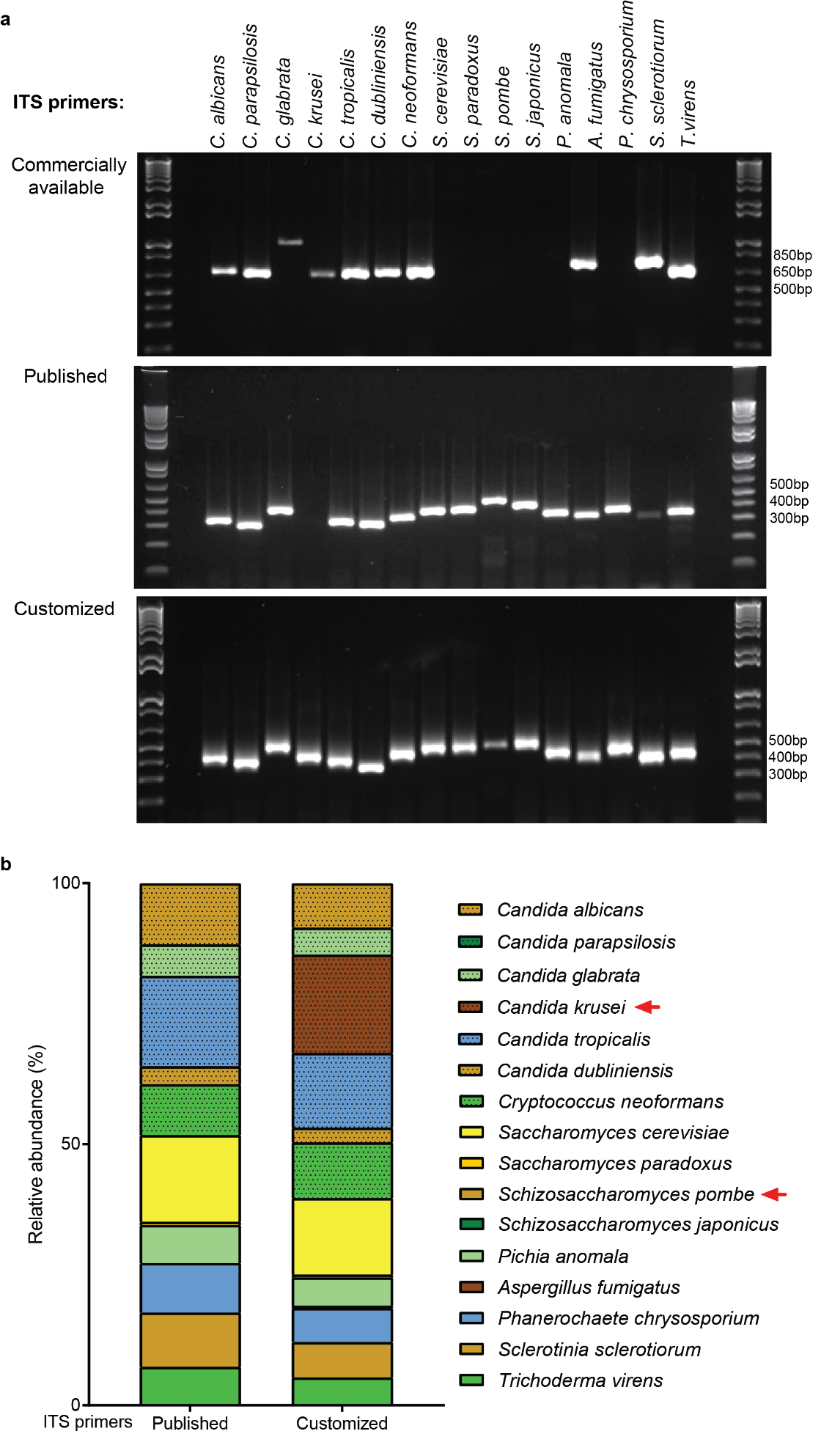
**

**Supplementary Figure 4. Customised ITS primers increase number of identified species.**

(**a**) PCR was performed on gDNA templates from 16 different fungal species with the Bioo Scientific commercial kit designed to amplify a region containing ITS1, 5.8S and ITS2 (top), with published primers targeting the ITS2 region only ^4^ (middle), or with our customized primers modified from the published primers (bottom). (**b**) An equimolar mix of gDNA from the same 16 fungal species was used as a template for ITS amplicon sequencing using either the published or our customised primers. Red arrows indicate species not detected by the published primers in the artificial community.


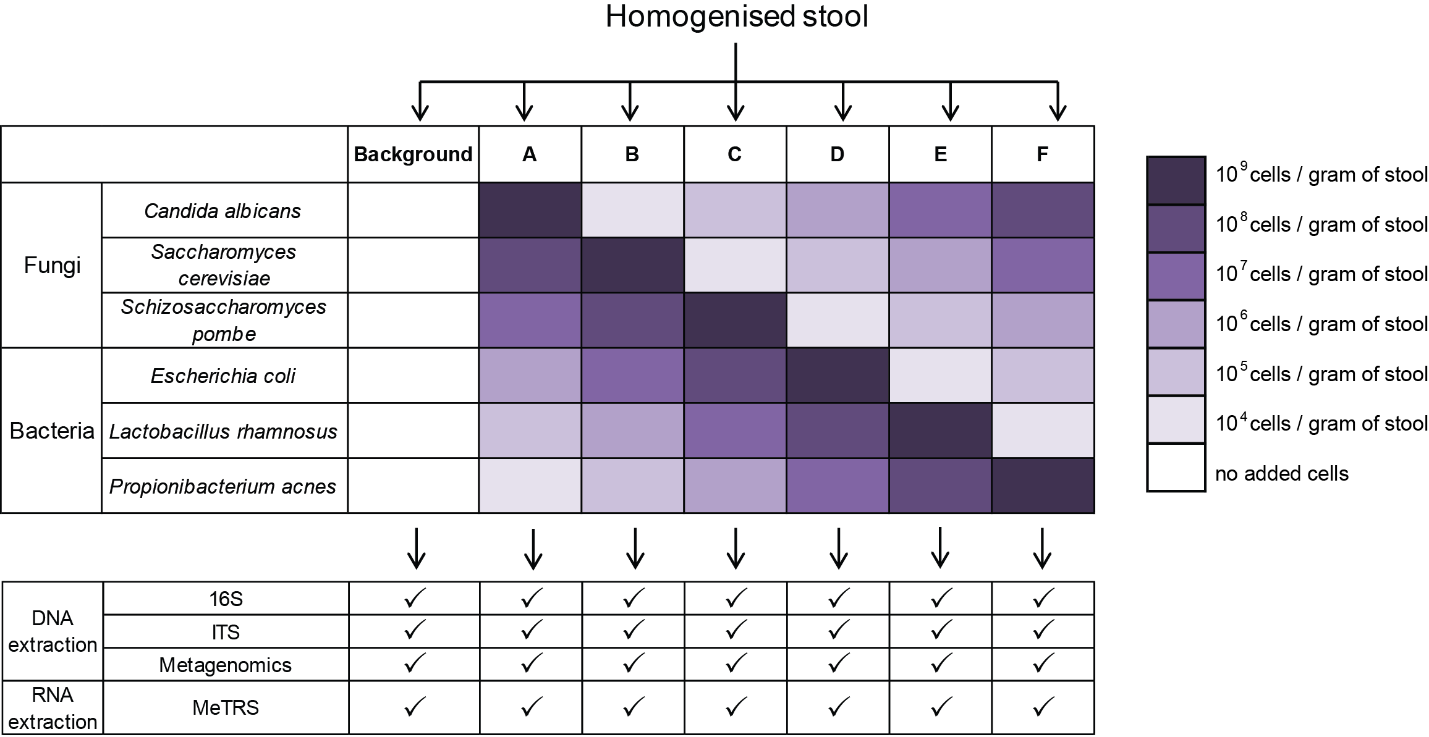


**Supplementary Figure 5. Latin square experimental design.**

Schematic representation of the experimental workflow. Logarithmically growing cells of indicated species were spiked into a homogenised stool sample according to the legend, before being aliquoted for DNA and RNA extraction. Genomic DNA was then used for metagenomics, 16S or ITS sequencing. RNA was processed for MeTRS. All sequencing runs were performed on HiSeq 2500 to generate 250bp paired-end reads.


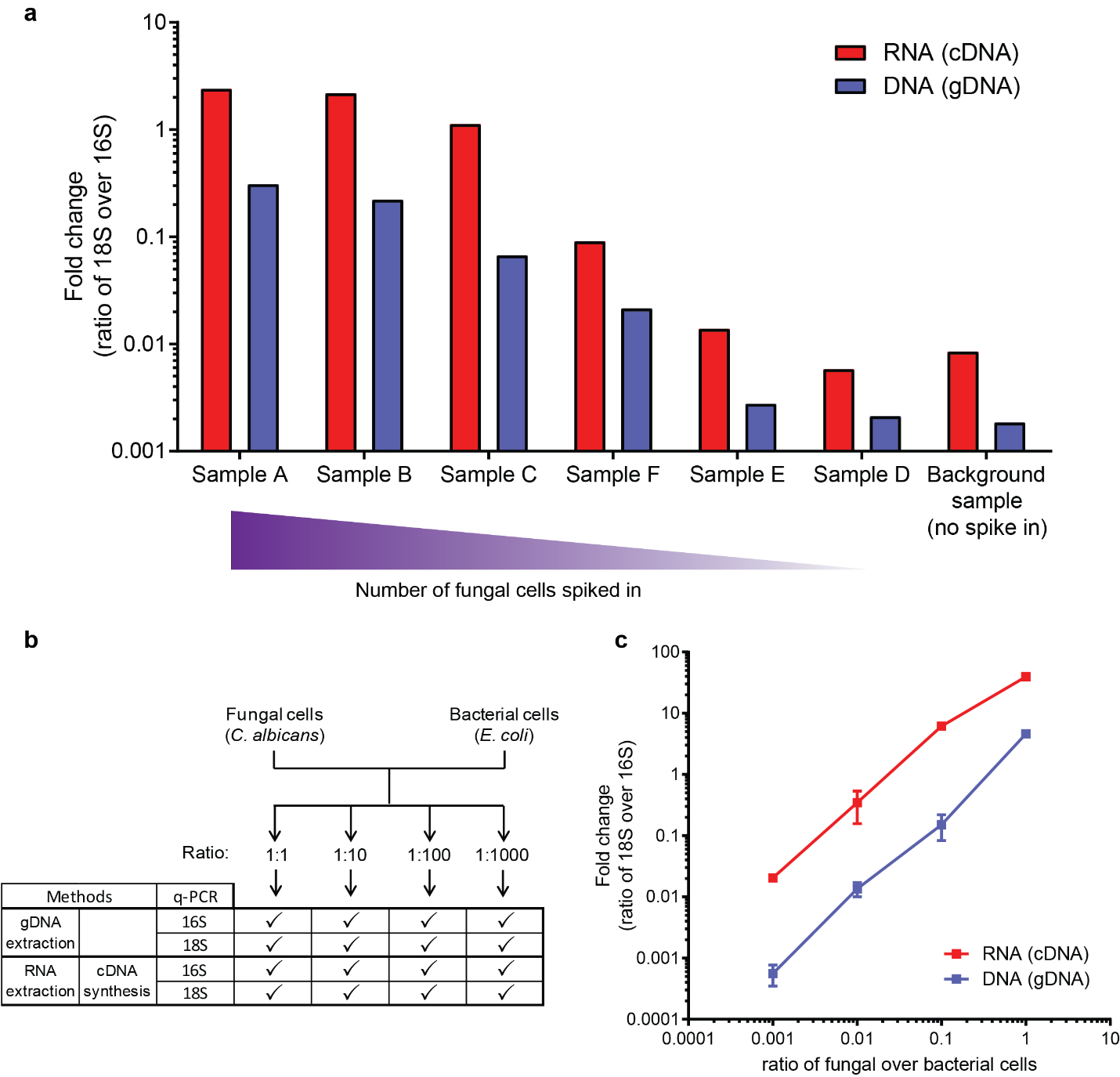


**Supplementary Figure 6. Fungi contain relatively more RNA than bacteria on a per cell basis.**

(**a**) Using qRT-PCR with primers targeted against 16S (BactQuant) or 18S (FungiQuant) rRNA regions, fold changes were measured using either gDNA or cDNA (RNA) samples from the Latin Square experiment (Figure S5). Samples are organised from highest to lowest amount of fungal cells spiked in. (**b**) Logarithmically growing cells of *C. albicans* and *E. coli* were mixed at the indicated ratios before being aliquoted for DNA and RNA extraction. (**c**) Genomic DNA and cDNA from experiment in (**b**) were used as template for qRT-PCR. The measured ratio of 18S over 16S copies is plotted against the nominal ratio of fungal cells over bacterial cells present in the sample.

**
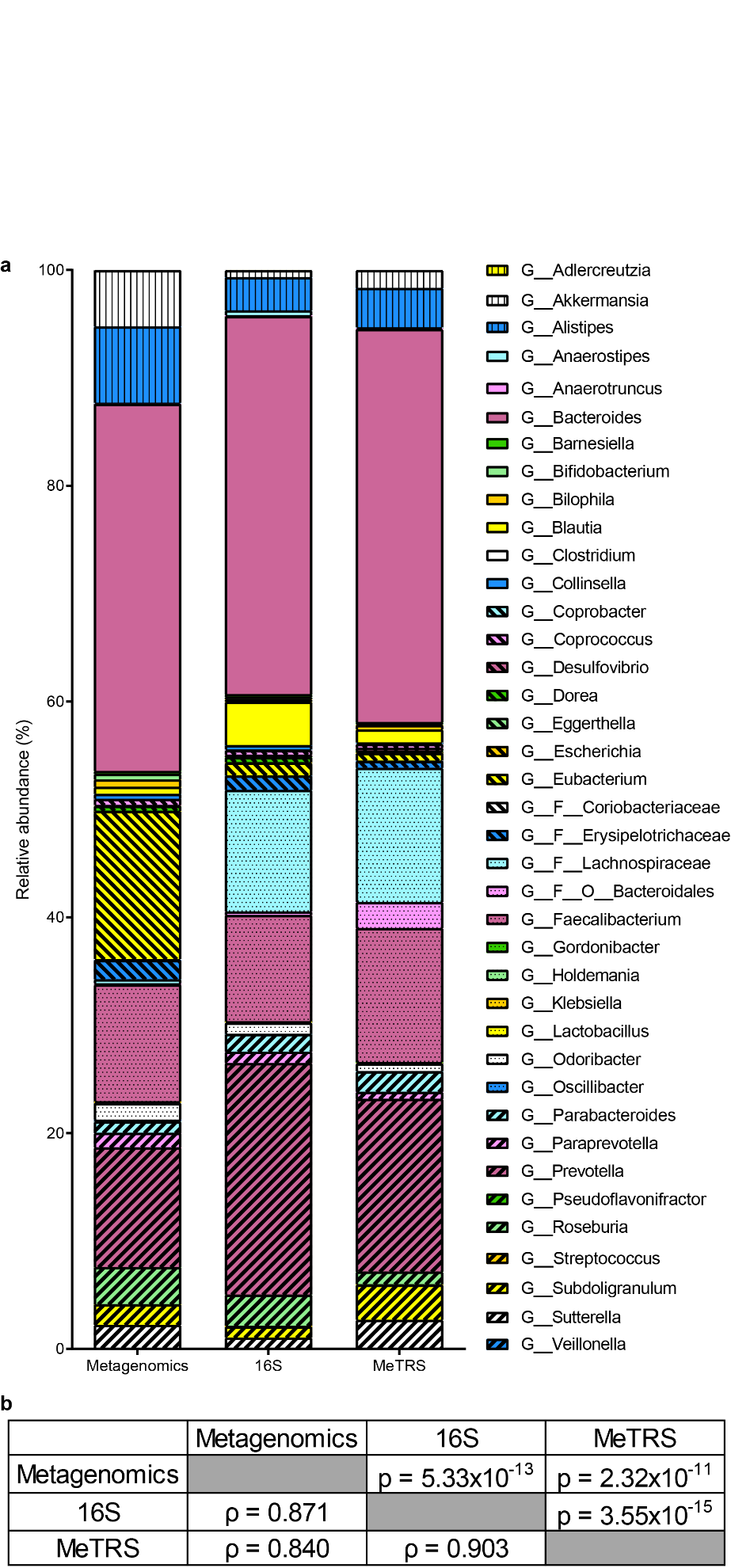
**

**Supplementary Figure 7. Genera distribution in metagenomics, 16S and MeTRS.**

(**a**) Relative abundance of the 39 genera commonly detected by all 3 technologies (metagenomics, 16S and MeTRS) in the background sample. (**b**) Spearman’s rank correlation and p-value between all three microbiota compositions.

**Supplementary References**

1 Bremer, H. & Dennis, P. P. Modulation of Chemical Composition and Other Parameters of the Cell at Different Exponential Growth Rates. *EcoSal Plus* **3**, doi:10.1128/ecosal.5.2.3 (2008).

2 Baker, G. C., Smith, J. J. & Cowan, D. A. Review and re-analysis of domain-specific 16S primers. *J Microbiol Methods* **55**, 541-555 (2003).

3 Teske, A. & Sorensen, K. B. Uncultured archaea in deep marine subsurface sediments: have we caught them all? *ISME J* **2**, 3-18, doi:10.1038/ismej.2007.90 (2008).

4 Ihrmark, K. *et al.* New primers to amplify the fungal ITS2 region--evaluation by 454-sequencing of artificial and natural communities. *FEMS Microbiol Ecol* **82**, 666-677, doi:10.1111/j.1574-6941.2012.01437.x (2012).

5 White, T. J., Bruns, T., Lee, S. & Taylor, J. in *PCR Protocols: A Guide to Methods and Applications* (eds M.A. Innis, D.H. Gelfland, J.J. Sninsky, & T.J. White) Ch. 38, 315-322 (Academic Press, 1990).

6 Liu, C. M. *et al.* BactQuant: an enhanced broad-coverage bacterial quantitative real-time PCR assay. *BMC Microbiol* **12**, 56, doi:10.1186/1471-2180-12-56 (2012).
